# A novel monoclonal antibody efficiently blocks the infection of serotype 4 fowl adenovirus by targeting fiber-2

**DOI:** 10.1186/s13567-018-0525-y

**Published:** 2018-03-09

**Authors:** Ping Wang, Jianjun Zhang, Weikang Wang, Tuofan Li, Guangchen Liang, Hongxia Shao, Wei Gao, Aijian Qin, Jianqiang Ye

**Affiliations:** 1 Key Laboratory of Jiangsu Preventive Veterinary Medicine, Key Laboratory for Avian Preventive Medicine, Ministry of Education, College of Veterinary Medicine, Yangzhou University, Yangzhou, 225009 Jiangsu China; 2 Jiangsu Co-innovation Center for Prevention and Control of Important Animal Infectious Diseases and Zoonoses, Yangzhou, 225009 Jiangsu China; 3 Joint International Research Laboratory of Agriculture and Agri-Product Safety, The Ministry of Education of China, Yangzhou University, Yangzhou, 225009 Jiangsu China; 4 Sinopharm Yangzhou VAC Biological Engineering Co. Ltd, Yangzhou, 225127 Jiangsu China; 5 Institutes of Agricultural Science and Technology Development, Yangzhou University, Yangzhou, 225009 Jiangsu China

## Abstract

A recent outbreak of hepatitis–hydropericardium syndrome caused by serotype 4 fowl adenovirus (FAdV-4) has resulted in significant economic losses to the poultry industry worldwide. However, little is known about the molecular pathogenesis of FAdV-4. In this study, a novel monoclonal antibody (mAb) targeting the fiber-2 protein of FAdV-4 was generated, mAb 3C2. Indirect immunofluorescence assay showed that mAb 3C2 neither reacted with serotype 8 fowl adenovirus (FAdV-8) nor reacted with the fiber-1 protein of FAdV-4; it specifically reacted with the fiber-2 protein of FAdV-4. Notably, mAb 3C2 could efficiently immunoprecipitate the fiber-2 protein in chicken liver cells either infected with FAdV-4 or transfected with pcDNA3.1-Fiber2. Moreover, mAb 3C2 demonstrated marked neutralizing activity against FAdV-4 and could efficiently inhibit the infection of FAdV-4 in vitro. Using truncated fiber-2 constructs, the epitope recognized by mAb 3C2 was determined to be located between amino acids 416–448 at the C-terminus of fiber-2. Our data not only provide a foundation for the establishment of a rapid fiber-2 peptide-based diagnostic assay for FAdV-4 but also highlight the critical role of the fiber-2 protein in mediating infection by FAdV-4. Furthermore, the epitope recognized by 3C2 might serve as a novel target for the development of a vaccine targeting FAdV-4.

## Introduction

Fowl adenovirus (FAdV) is a member of the family *Adenoviridae*, genus *Aviadenovirus* [1]. To date, five species (FAdV-A to FAdV-E) and 12 serotypes (FAdV-1 to 8a and 8b to 11) of FAdV have been identified by cross-neutralization assay and genome analysis [2]. FAdV infection generally causes subclinical symptoms in infected chickens; however, acute infections can result in inclusion body hepatitis (IBH), hepatitis–hydropericardium syndrome (HPS), and gizzard erosion and ulceration (GEU) [2–4]. Among the 12 serotypes, FAdV-2, 8a, 8b and 11 generally cause IBH, whereas FAdV-4 is the main cause of endemic HPS [5–8]. Notably, HPS caused by FAdV-4 is a major killer in chicken flocks [9]. Recently, an outbreak of FAdV-4 reached epidemic proportions, possibly due to enhanced virulence, causing massive economic losses in the poultry industry [2, 3, 6, 10–13]. Notably, the mortality caused by endemic FAdV-4 in China has reached as high as 80% in several domestic chicken flocks [13–16]. Moreover, sequencing analysis revealed that recent Chinese FAdV-4 isolates carried unique mutations with significant deletions in their genome compared with FAdV-4 isolates from other countries and regions [13, 14]. However, little is known about the molecular mechanism underlying the infection and pathogenesis of FAdV-4.

Among the structural proteins encoded by adenovirus, the fiber protein plays vital roles in mediating viral infection and determining the antigenicity [17–19]. In contrast to other adenoviruses, FAdV-1, FAdV-4 and FAdV-10 encode two fiber proteins, designated fiber-1 and fiber-2 [17]. Although a previous report showed that recombinant fiber-2 protein could provide better protection against FAdV-4 than recombinant fiber-1 [20], the roles of the two fiber proteins in the infection and pathogenesis of FAdV-4 are unclear. Due to the lack of any monoclonal antibody (mAb) specific for the fiber proteins of FAdV-4, the progression of such studies has been severely limited. In this study, a novel mAb specific to the fiber-2 protein of FAdV-4 (designated mAb 3C2) was generated, and its epitope was identified. mAb 3C2 could not only immunoprecipitate the fiber-2 protein in infected cells but also blocked the infection of FAdV-4 in vitro.

## Materials and methods

### Viruses, cells and plasmids

FAdV-4 isolate SD2015 and FAdV-8 isolate SQ2015 were isolated and maintained in our laboratory [13]. The chicken liver cell line (LMH) was purchased from ATCC and cultured in F12/DMEM (Gibco, NY, USA) supplemented with 10% FBS (Lonsera, Shanghai, China). Plasmids pcDNA3.1-F1 and pcDNA3.1-F2 expressing fiber-1 and fiber-2 of FAdV-4, respectively, were generated in our laboratory.

### Antibodies

Chicken sera against FAdV-4 and FAdV-8 were generated through vaccination of 14-day-old SPF chickens with the corresponding inactivated viruses, provided by Sinopharm Yangzhou VAC Biological Engineering Co., Ltd. Monoclonal antibody 6E6 against HA of H9N2 was developed in our laboratory [21]. FITC-labelled goat anti-mouse IgG, HRP-labelled goat anti-mouse IgG and HRP-labelled rabbit anti-chicken IgY(H + L) were purchased from Sigma (CA, USA).

### Generation of mAbs

10^7^ TCID_50_ of FAdV-4 isolate SD2015 was used to immunize 6-week-old BALB/C mice four times every 10 days. At day 4 following the fourth immunization, the splenic cells from one immunized mouse were collected and fused with SP2/0 cells with PEG1500 (Roche, Mannheim, Germany), as previously described [22]. After culture with HAT-selective medium (Sigma), hybridoma cells secreting antibodies against the fiber-2 protein of FAdV-4 were screened using ELISA coating with purified GST-Fiber2 (generated in our laboratory). After sub-cloning of the positive hybridoma cells, the characteristics of mAbs secreted by these positive clones were identified through immunofluorescence assay, ELISA, immunoprecipitation and neutralization testing. The isotype of mAb was determined with a mouse mAb isotyping kit (Thermo Scientific, Massachusetts, USA) according to the manufacturer’s protocol. The mAbs in ascites were generated as previously described and purified using protein G columns (GE Healthcare Life sciences, Uppsala, Sweden) [23].

### ELISA

The purified GST-Fiber2 protein (generated in our laboratory) was used to coat the wells of plates [0.36 μg protein in each well) at 4 °C overnight. The ELISA plates were washed with PBST (0.01 M phosphate-buffered saline (PBS), pH 7.2, 0.05% Tween 20] once and then blocked with PBST containing 5% skim milk for 1 h. After washing once, the supernatant of the hybridoma cells or serial dilutions of mAbs were added and incubated for 1 h at 37 °C. After three washes with PBST, the plates were supplemented with 100 μL of a 1:10 000 dilution of HRP-labelled goat anti-mouse IgG. After 30 min at 37 °C, the ELISA plates were washed three times with PBST, and then supplemented with 100 μL TMB (Solarbio, Beijing, China) for development. After 10 min at 37 °C, the development reaction was stopped by adding 50 μL of 2 M H_2_SO_4_, and the absorbance values at 450 nm (OD450) were measured with a Microplate Reader (TECAN Infinite M200 Pro). When the OD450 value of the sample was greater than 0.2, the sample was regarded as positive.

### Indirect immunofluorescence assay (IFA)

LMH cells were infected with 1000 TCID_50_ of FAdV-4 SD2015 and FAdV-8 SQ2015 or transfected with pcDNA3.1-F1 and pcDNA3.1-F2, respectively. After day 3 post-infection or transfection, the cells were fixed with chilled acetone and ethanol (3:2) for 5 min. mAbs at the indicated dilutions were incubated with the fixed cells for 45 min. After three washes with PBS, FITC-conjugated secondary antibodies at 1:150 dilutions were incubated with the cells for another 45 min. After three washes with PBS, the plates were observed with an inverted fluorescence microscope.

### Immunoprecipitation and immunoblotting

LMH cells infected with 1000 TCID_50_ of FAdV-4 SD2015 or transfected with pcDNA3.1-F2 for 3 days were collected and lysed in lysis buffer (CST, MA, USA) with PMSF (Beyotime, Shanghai China), protease and phosphatase inhibitors (CST). Lysates were then mixed and immunoprecipitated with 2 μg mAb at 4 °C overnight. The next day, 40 μL of protein G-Sepharose beads (Beyotime) were added to the mixture and incubated at 4 °C for 3 h. Then, the mixture of proteins and beads was washed five times with PBS, and the immunoprecipitated proteins were eluted by boiling with loading buffer. After separation via SDS-PAGE, the denatured samples were transferred onto nitrocellulose membranes (NCs) (GE Healthcare Life sciences, Freiburg, Germany) for Western blot analysis. After blocking with skim milk in PBST for 1 h at room temperature (RT), the NCs were incubated with chicken sera against FAdV-4 (1:1000 dilution in PBST) for 2 h at RT. After three washes with PBST, the NCs were incubated with HRP-conjugated rabbit anti-chicken IgG (1:10 000 dilution in PBST). After three washes, the NCs were developed using a fully automatic chemiluminescence image analysis system (Tanon 5200).

### Neutralization testing

Serial dilutions of mAbs were first mixed with 1000 TCID_50_ of FAdV-4 isolate SD2015 in equal volumes and incubated for 1 h at 37 °C. Then, the mixture was inoculated into fresh LMH cells. After incubation for 2 h, the inoculated supernatant was discarded, and the inoculated cells were washed once with PBS. The inoculated cells were then maintained in DMEM/F12 medium with 1% FBS for 3 days. Then, the viral replication in these inoculated cells was analysed through IFA and Western blot, as described above.

### Epitope mapping

To map the epitopes recognized by mAb 3C2, serial fiber-2 recombinant constructs with various deletions at the C-terminus were generated with a ClonExpress II One Step Cloning Kit (Vazyme Biotech, Nanjing, China) as previously described. The primers used for amplifying the fiber-2 gene with different deletions and the linear pcDNA3.1 vector are listed in Table 1. LMH cells transfected with the different fiber-2 recombinants were fixed at day 2 post-transfection and stained with mAb 3C2 through IFA, as described above.

**Table 1:** **Primers for amplifying the truncated fiber2 gene and the linear pcDNA3.1**

PCR product	Primer sequence (5′–3′)
F2_1-415aa	Forward: AGCTTGGTACCGAATGCTCCGGGCCCCTAA
	Reverse: ATATCTGCAGAATTTACTATAGCATAGAAG
F2_1-382aa	Forward: AGCTTGGTACCGAATGCTCCGGGCCCCTAA
	Reverse: ATATCTGCAGAATTTAGGCTGAACACTTGG
F2_1-349aa	Forward: AGCTTGGTACCGAATGCTCCGGGCCCCTAA
	Reverse: ATATCTGCAGAATTTACGTGAGGGTGGCGG
F2_1-316aa	Forward: AGCTTGGTACCGAATGCTCCGGGCCCCTAA
	Reverse: ATATCTGCAGAATTTATTGGCGGAGTTGAG
F2_1-283aa	Forward: AGCTTGGTACCGAATGCTCCGGGCCCCTAA
	Reverse: ATATCTGCAGAATTTAGGTAGTAGGCGCAA
F2_1-250aa	Forward: AGCTTGGTACCGAATGCTCCGGGCCCCTAA
	Reverse: ATATCTGCAGAATTTAGGAGACGCTCCCCC
F2_1-217aa	Forward: AGCTTGGTACCGAATGCTCCGGGCCCCTAA
	Reverse: ATATCTGCAGAATTTAGGTCCGCTGGGATC
F2_1-182aa	Forward: AGCTTGGTACCGAATGCTCCGGGCCCCTAA
	Reverse: ATATCTGCAGAATTTACCTGCGCTTTTAGG
Linear pcDNA3.1	Forward: GAATTCTGCAGATATCCAGCACAGTG
	Reverse: GCTCGGTACCAAGCTTAAGTTTAAACG

## Results

### Generation of a novel monoclonal antibody against fiber-2 of FAdV-4

**Figure 1:**
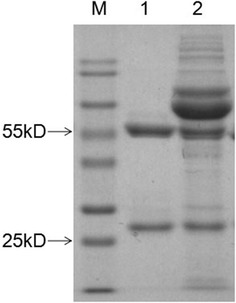
**SDS-PAGE analysis of the purified 3C2.** 3C2 ascites were purified with protein G and analysed by SDS-PAGE. Lane M: Protein Marker Blue Plus II; lane 1: purified 3C2; lane 2: non-purified 3C2.

**Figure 2:**
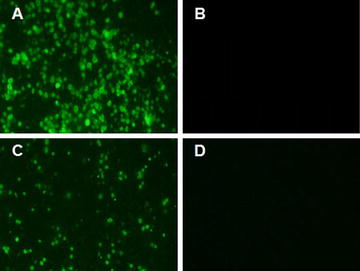
**Specificity analysis for 3C2 by IFA.** LMH cells were infected with either FAdV-4 or FAdV-8 or transfected with pcDNA3.1-F1 or pcDNA3.1-F2. Then, 3C2 was used as the primary antibody to perform IFA to analyse its specificity. **A**, **B** 3C2 reacted with chicken liver cells infected with FAdV-4 but not FAdV-8. **C**, **D** 3C2 reacted with LMH cells transfected with pcDNA3.1-F2 but not pcDNA3.1-F1.

To generate monoclonal antibodies specific to fiber-2 of FAdV-4, FAdV-4 isolate SD2015 was used to immunize BALB/C mice. After the fusion of splenic cells from immunized mice and SP2/0 cells, hybridoma cells secreting antibodies against the fiber-2 protein of FAdV-4 were screened by ELISA. In the ELISA, purified GST-Fiber2 was used as the coating antigen. Ultimately, one hybridoma clone showed strong positivity in ELISA screening and was designated mAb 3C2. After sub-cloning, ascites containing mAb 3C2 was generated as previously described, and then the characteristics of mAb 3C2 were tested. Analysis with a mAb isotyping kit revealed that the isotype of mAb 3C2 was IgG2b with a Kappa light chain. The ELISA titre of the ascites of mAb 3C2 could reach 1:1 280 000. mAb 3C2 was also purified using protein G, and only the heavy and light chain could be found in the purified mAb 3C2, as shown in Figure 1. Specificity analysis showed that mAb 3C2 only reacted with LMH cells infected with FAdV-4, not with FAdV-8 (Figure 2). Moreover, mAb 3C2 only reacted with LMH cells transfected with pcDNA3.1-Fiber2, not with pcDNA3.1-Fiber1 (Figure 2) in IFA.

### Immunoprecipitation of the fiber-2 protein of FAdV-4 with mAb 3C2

**Figure 3:**
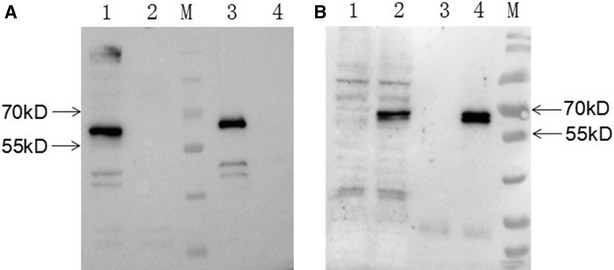
**Immunoprecipitation analysis.** LMH cells were infected with FAdV-4 or transfected with pcDNA3.1-F2. Then, 3C2 was used as the capture antibody to perform immunoprecipitation, followed by Western blot analysis using chicken sera against FAdV-4. **A** Lanes 1 and 2: lysates of LMH cells infected with or without FAdV-4, respectively. Lanes 3 and 4: 3C2-immunoprecipitated pellets of LMH cells infected with or without FAdV-4, respectively. **B** Lanes 1 and 2: lysates of LMH cells transfected without or with pcDNA3.1-F2, respectively. Lanes 3 and 4: 3C2-immunoprecipitated pellets of LMH cells transfected without or with pcDNA3.1-F2, respectively.

Although mAb 3C2 could efficiently recognize the fiber-2 protein in ELISA and IFA analyses, mAb 3C2 could not recognize the fiber-2 protein via Western blot under induced conditions. This indicated that the epitope recognized by mAb 3C2 was likely to be dependent on structure. To further determine whether mAb3 C2 could bind the fiber-2 protein in cell lysates, immunoprecipitation assays were performed. As shown in Figures 3A and B, the purified mAb 3C2 could efficiently immunoprecipitate the fiber-2 protein in LMH cells either infected with FAdV-4 isolate SD2015 or transfected with pcDNA3.1-Fiber2. The immunoprecipitation assay results suggested that mAb 3C2 could be used for identifying the proteins or signaling pathways that the fiber-2 protein interacts with, thereby elucidating the roles of fiber-2 in the pathogenesis of FAdV-4.

### Inhibition of FAdV-4 infection by mAb 3C2

**Figure 4:**
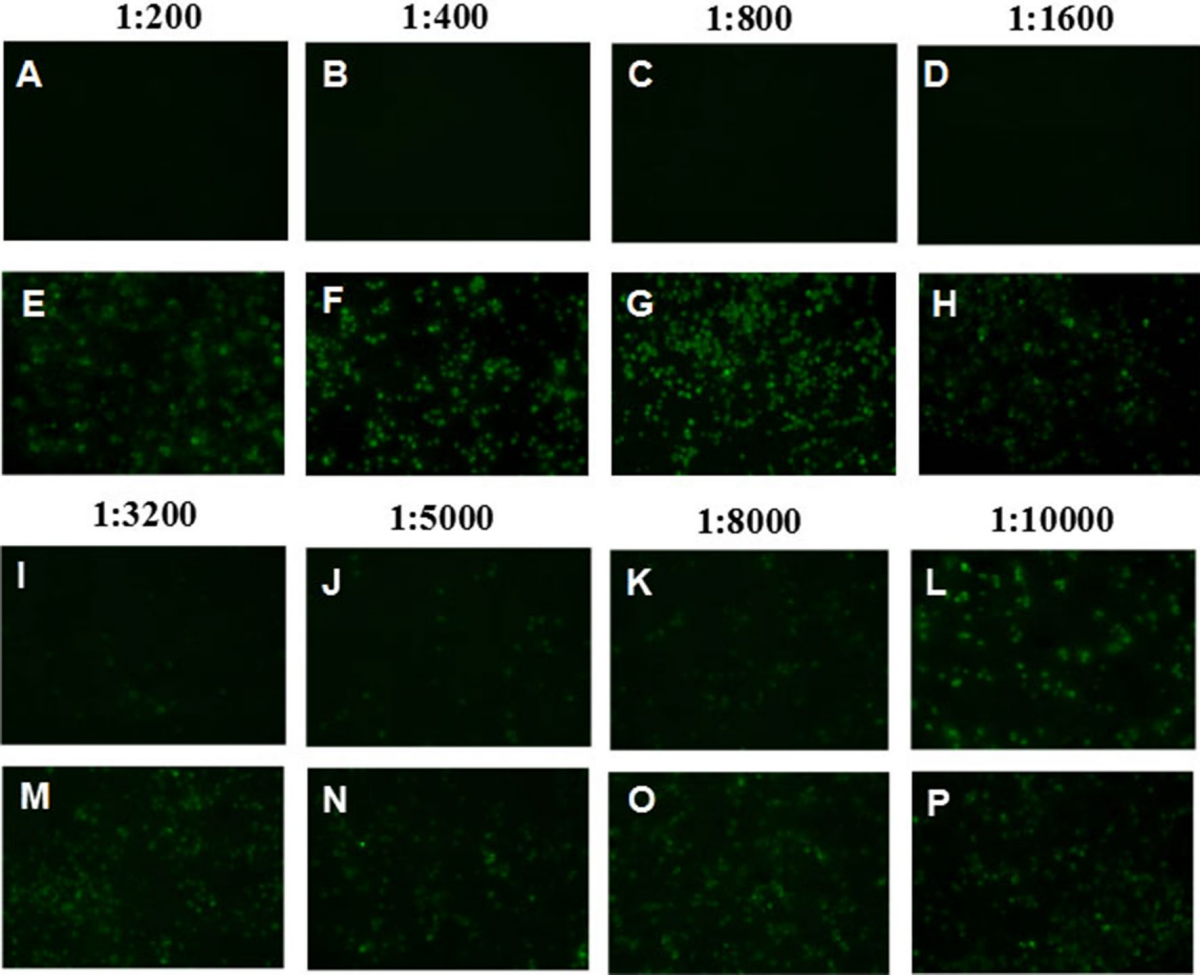
**IFA analysis for the neutralization activity of 3C2.** Serial dilutions of 3C2 or control 6E6 mAbs were first mixed with FAdV-4, and then, LMH cells were infected with the mixture and analysed by IFA, as described in “Materials and methods”. **A**–**D** and **I**–**L** LMH cells infected with the mixture of virus and serial dilutions of 3C2; **E**–**H** and **M**–**P** LMH cells infected with the mixture of virus and serial dilutions of mAb 6E6.

**Figure 5:**
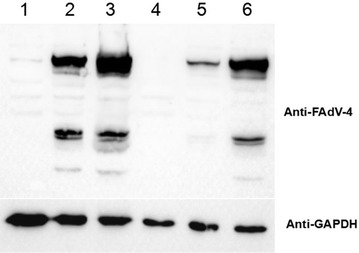
**Western blot analysis of the neutralization activity of 3C2.** 1:200 and 1:5000 dilutions of 3C2 or control 6E6 mAbs were first mixed with FAdV-4, and then LMH cells were infected with the mixture and analysed by Western blot using chicken sera against FAdV-4, as described in “Materials and methods”. Lanes 1 and 2: lysates of LMH cells infected with the mixture of virus and the 1:200 dilutions of 3C2 and 6E6, respectively. Lanes 3 and 4: lysates of LMH cells infected with or without FAdV-4, respectively. Lanes 5 and 6: lysates of LMH cells infected with the mixture of virus and 1:5000 dilutions of 3C2 and 6E6, respectively.

Since the fiber-2 protein is located on the FAdV-4 viral surface, it might play vital roles in mediating viral infection. To evaluate this hypothesis, we determined whether mAb 3C2 could block or inhibit the infection or replication of FAdV-4 in vitro. As shown in Figure 4, mAb 3C2 could efficiently block FAdV-4 infection in a dose-dependent manner in neutralization tests. The viral replication of FAdV-4 could not be identified in cells treated with mAb 3C2 at dilutions of 1:200 to 1:800, whereas FAdV-4 replicated efficiently in cells treated with a control antibody (6E6). Notably, mAb 3C2 treatment at dilutions of 1:1600 to 1:5000 still showed efficient blockade of FAdV-4 infection. To further demonstrate the neutralizing activity of mAb 3C2, Western blot analysis was used to measure the levels of FAdV-4 viral proteins in cells treated with mAb 3C2 at dilutions of 1:200 and 1:5000. As described in Figure 5, virus-specific proteins could not be detected in FAdV-4-infected cells with treatment of mAb 3C2 at a dilution of 1:200, whereas these viral proteins could be found in FAdV-4-infected cells treated with a control antibody or without any antibody treatment. Moreover, only modest levels of viral proteins could be found in infected cells treated with mAb 3C2 at a dilution of 1:5000. The neutralizing activity of mAb 3C2 against FAdV-4 indicates that the fiber-2 protein plays vital roles in mediating the infection of FAdV-4.

### Mapping the epitope in fiber-2 recognized by mAb 3C2

**Figure 6:**
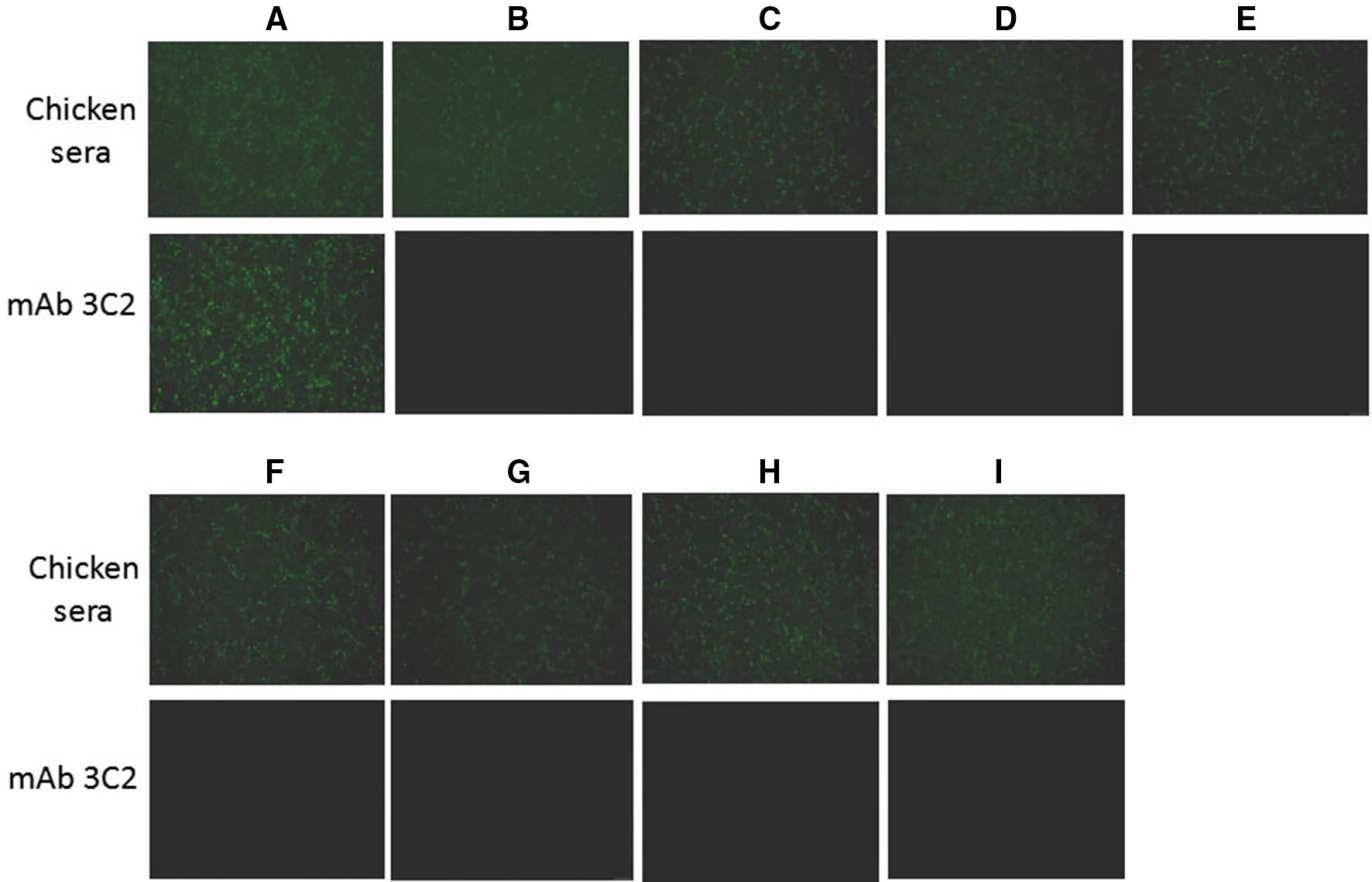
**Epitope mapping for mAb 3C2 using truncated fiber-2 constructs.** LMH cells transfected with the different truncated fiber-2 constructs respectively were fixed, and stained with mAb 3C2 or chicken sera against FAdV-4 against FAdV-4 through IFA. **A**–**I** The LMH cells were transfected with pcDNA3.1-F2, F2_1-415aa, F2_1-382aa, F2_1-349aa, F2_1-316aa, F2_1-283aa, F2_1-250aa, F2_1-217aa and F2_1-182aa respectively.

Since mAb 3C2 reacted with a GST-F2_181-448aa fusion protein, but not with a GST-F2_1-180aa fusion protein by ELISA (data not shown), the epitope recognized by mAb 3C2 was located in the C-terminus of F2_181-448aa. Therefore, to determine the exact neutralizing epitope in fiber-2 recognized by mAb 3C2, different truncated fiber-2 recombinant constructs were transfected into LMH cells, and IFA was performed. As shown in Figure 6, chicken polyclonal antibodies against FAdV-4 could react robustly with LHM cells transfected with all the different truncated fiber-2 constructs, whereas mAb 3C2 only reacted with LHM cells transfected with full-length fiber-2, not with these truncated fiber-2 constructs. This demonstrated that the epitope recognized by mAb 3C2 was located at amino acids 416–448 in the C-terminus of the fiber-2 protein. Since mAb 3C2 had high neutralizing activity, the epitope at 416–448aa that reacted with mAb 3C2 could serve as a novel peptide for designing diagnostic tools or vaccine preparation.

## Discussion

Among the 12 serotypes of FAdV, only serotypes FAdV-1, FAdV-4 and FAdV-10 express two fiber proteins, fiber-1 and fiber-2. Although the vital role of the fiber proteins of FAdV-1 (CELO strain) in viral infection and replication have been demonstrated using CELO mutants expressing truncated fiber-1 and fiber-2 proteins, the roles of the fiber proteins of FAdV-4 have not been elucidated. In this study, the critical role of fiber-2 of FAdV-4 has been demonstrated by the blockade of infection using a novel mAb 3C2 targeting fiber-2.

A previous report by Schachner et al. demonstrated that fiber-2, but not fiber-1, could provide efficient protection against lethal challenge with FAdV-4 [20]. However, neutralizing antibodies in protected chickens prior to challenge could not be efficiently detected by Schachner et al., which indicated that either the fiber-2 expressed by Schachner et al. could not efficiently induce neutralizing antibodies or the neutralizing epitope of the expressed fiber-2 could not be efficiently targeted by the immune cells. The mAb 3C2 against fiber-2 generated here could efficiently block infection by FAdV-4, highlighting that the epitope recognized by mAb 3C2 may be a vital neutralizing epitope. Through the construction of truncated fiber-2 constructs, the neutralizing epitope recognized by mAb 3C2 was identified to be located between aa 416–448 in the fiber-2 protein of FAdV-4. Sequence analysis revealed that four amino acids (at positions 422, 428, 445 and 447) in the epitope showed variation among the different FAdV-4 isolates from the NCBI database. Notably, 47.8% of FAdV-4 isolates carry an N residue at position 428, which provides a potential glycosylation site. The FAdV-4 isolate SD2015 used in this study also has this glycosylation site at position 428. However, the role of this potential glycosylation site at position 428 in the antigenicity and pathogenesis of FAdV-4 remains to be further investigated.

In summary, this is the first demonstration of a novel mAb, 3C2, targeting the fiber-2 protein of FAdV-4 with both IP and neutralization activity. mAb 3C2 specific to the fiber-2 protein could efficiently block infection by FAdV-4, providing strong evidence that the fiber-2 protein plays vital roles in mediating FAdV-4 infection, possibly through binding to its cell receptor. Fiber-2-interacting proteins (including the cell receptor) are currently being identified using mAb 3C2 through Co-IP and Mass Spectrometry analyses. In addition, the mAb 3C2 generated here and its identified epitope may be useful for the development of passive immunotherapy and rapid diagnostic tests, such as antigen-capture ELISA or peptide-based ELISA.
